# Association of pelvic inflammatory disease (PID) with ovarian cancer: a nationwide population-based retrospective cohort study from Taiwan

**DOI:** 10.1186/s12905-021-01413-2

**Published:** 2021-07-28

**Authors:** Cherry Yin-Yi Chang, Kent Yu-Hsien Lin, Chien-Chu Huang, Wu-Chou Lin

**Affiliations:** 1 Department of Obstetrics and Gynecology, China Medical University Hospital, Taichung, Taiwan; 2 Department of Medicine, China Medical University, No. 2, Yuh-Der Road, Taichung, 404 Taiwan; 3 Department of Obstetrics and Gynaecology, Women and Children’s Health, Royal North Shore Hospital, Sydney, NSW Australia; 4 Graduate Institution of Biomeidcal Sciences, China Medical University, No. 2, Yuh-Der Road, Taichung, 404 Taiwan

## Abstract

**Background:**

Pelvic inflammatory disease (PID) is an important health issue for women. Infection and inflammation play an important role in carcinogenesis and PID has been reported to be associated with ovarian cancer in some small scale studies.

**Aim:**

We sought to determine whether PID is associated with an elevated risk of ovarian cancer in Asian women.

**Methods:**

Using data from Taiwan’s National Health Insurance Research Database (NHIRD), our retrospective cohort study included women diagnosed with PID (cases) between the years of 2000 till 2012. Each case was matched with two women without PID (controls) by age and the year of first entry into the database. Both study cohorts were followed-up until the first event of ovarian cancer, withdrawal from the NHI program, death, or the end of the study period (December 31, 2012). Cox proportional hazards regression models were used to estimate crude and adjusted hazard ratios (HRs and aHRs) with their corresponding 95% confidence intervals (95% CIs) for the association of PID and ovarian cancer risk, with and without adjusting for potential confounders.

**Results:**

During an approximate 10 years of follow-up, cases were significantly more likely than controls to develop ovarian cancer (incidence rates of 0.27 and 0.16 per 1,000 person-years, respectively; *P* < 0.001). Women with a history of PID had a 1.49-fold elevated risk for ovarian cancer (aHR, 1.49; 95% CI, 1.21–1.84; *P* < 0.001).

**Conclusion:**

Our study evidence supports the contention that PID increases the risk of developing ovarian cancer among Taiwanese women. Gynecologists should undertake careful assessments and closely follow patients with PID, who are at long-term risk of developing ovarian cancer. Our findings need further verification in other international cohorts.

**Supplementary Information:**

The online version contains supplementary material available at 10.1186/s12905-021-01413-2.

## Introduction

Pelvic inflammatory disease (PID) is caused by sexually transmitted infections at various sites in the female genital tract and affects the upper part of the female reproductive system, i.e., the uterus, ovaries, and fallopian tubes, as well as other pelvic organs and even abdominal organs [1]. PID is often diagnosed among women of reproductive age. Its clinical features vary according to the patient’s immune and health status. Typically, there are no obvious symptoms and signs of PID until chronic inflammation develops in the pelvic cavity, which can lead to serious consequences, such as preterm labor [2], infertility [3], ectopic pregnancy [4], and pelvic organ adhesions [5]. Research has shown that infection and chronic inflammation play an important role in carcinogenesis [6–8].

Ovarian cancer has a high mortality rate, due to asymptomatic tumor growth and delayed onset of symptoms, with most patients was not diagnose until they experience signs or symptoms of back pain, fatigue, constipation, abdominal discomfort, postprandial fullness sensations and urinary symptoms (urgency or frequency) [9, 10]. Thus, almost two-thirds of patients are diagnosed with advanced disease at initial presentation [11]. Optimal treatment for advanced ovarian cancer consists of primary debulking surgery followed by platinum-based chemotherapy, although prognosis remains poor. Thus, early detection and identifying women at high risk of developing ovarian cancer are of crucial importance. Several risk factors are associated with ovarian cancer, including low parity, infertility, early age at menarche, and late age at menopause [12–15]. Recurrent inflammation may contribute to cancer cell formation. It is thought that chronic exposure to external or endogenous triggers of immunity and persistent immune cells injure the surrounding epithelium cells and that exposure to pathogens can lead to chronic inflammation that promotes tumor growth and progression [16].

Due to some cues in the literature implied about the association between PID and ovarian cancer risk, we examined data from Taiwan’s National Health Insurance Research Database (NHIRD) for this long-term population-based study, to examine the risk for developing ovarian cancer among women with PID.

## Materials and methods

### Data source

Taiwan launched universal health coverage in 1995 with its National Health Insurance program, which now includes over 99% of the population. Anonymized, de-identified patient data from the National Health Insurance Research Database (NHIRD) were obtained for this investigation. The NHIRD contains health insurance information covering medical procedures, prescribed medications, outpatient and inpatient records, and emergency department visits. This study was granted approval by China Medical University Hospital’s Review Board and Ethics Committee (CMUH number: CMUH-104-REC2-115 (CR-4)). The main data source is the Longitudinal Health Insurance Database 2000 (LHID2000), which contains details on medical care given to a randomly selected 1 million insured individuals in the NHIRD covering the period from 1996 through 2013. It is established that the distributions of age and sex ratios are similar between the LHID2000 and NHIRD [17].

### Study participants

**Fig. 1:**
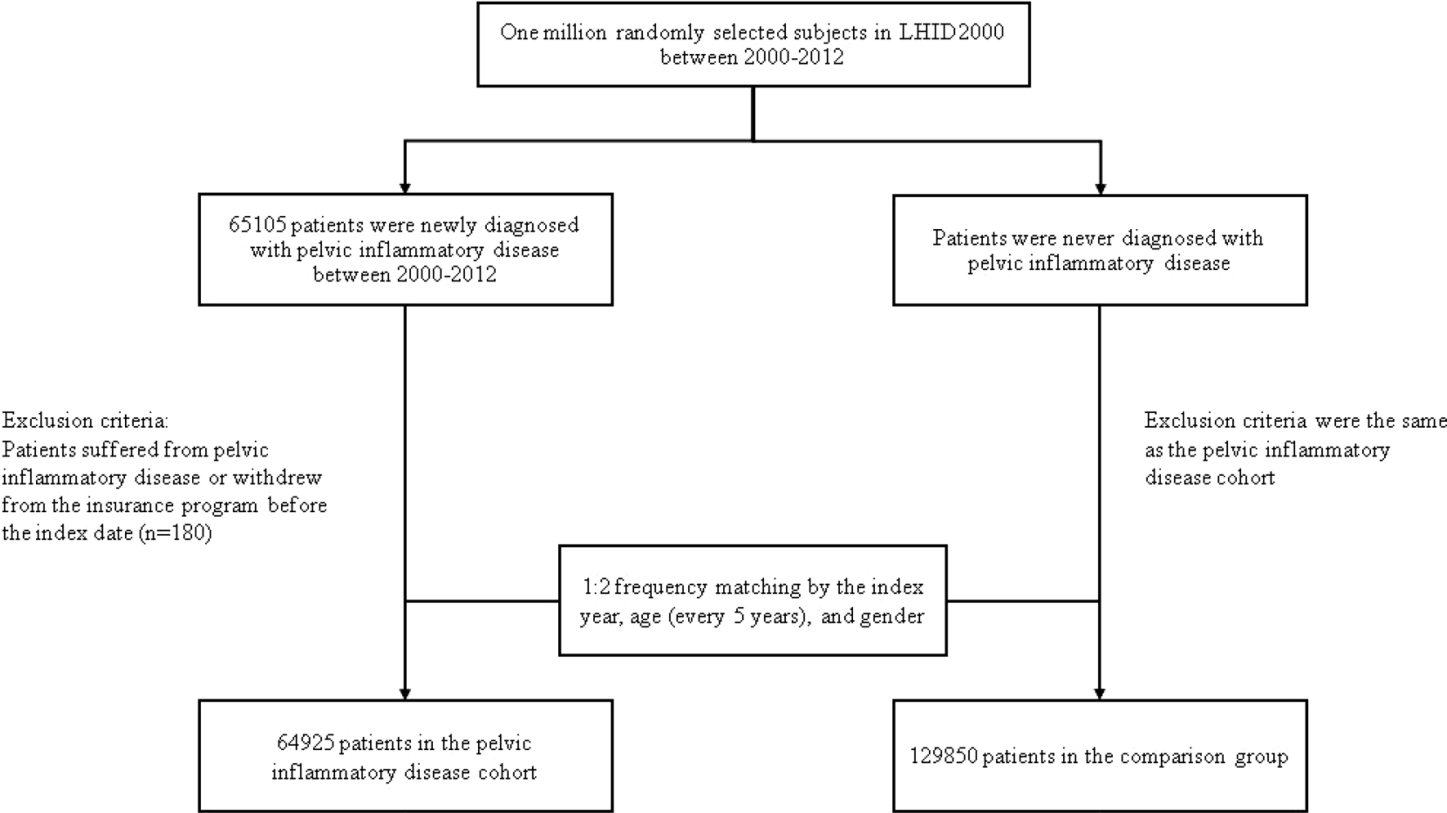
The flowchart of study sample selection from National Health Insurance Research Database in Taiwan

The study population comprised women aged 18 years and older diagnosed with PID according to the International Classification of Diseases, 9th revision, Clinical Modification (ICD-9-CM) code 614.3-614.9, between 2000 and 2012, who served as the case cohort. The date of the PID diagnosis was defined as the index date. Each case was matched with 2 women without PID (control group) by age and index date. The index data marked the commencement of follow-up. Both study cohorts were followed-up until the first event of ovarian cancer (ICD-9-CM code 183), withdrawal from the NHI program, death, or the end of the study period (December 31, 2012) (Fig. 1). Participants aged younger than 18 years were excluded from the study.

### Comorbidities

Common baseline comorbidities were identified by ICD-9 codes recorded in the LHID2000 prior to the index date and included Lynch syndrome (hereditary nonpolyposis colorectal cancer) and colon cancer (ICD-9-CM code 153.9) [18, 19], breast cancer (ICD-9-CM code 174.9) [20, 21], uterus cancer (ICD-9-CM codes 179 and 182) [20], rectum cancer (ICD-9-CM code 154), endometriosis (ICD-9-CM code 617) [22], infertility (ICD-9-CM code 628), and obesity (ICD-9-CM code 278). The criteria of obese defined by the Department of Health in Taiwan is BMI > or = 27 [23].

### Statistical analysis

Baseline characteristics between the PID cohort and controls were compared using the Chi-square test for categorical variables and the Wilcoxon rank-sum test for continuous data. The ages of the study cohorts were compared using the t-test.Ovarian cancer-free survival curves for the PID and control groups were calculated by Aalen-Johansen analysis and the difference was tested with the log-rank test. Cox proportional hazards regression models were used to estimate crude and adjusted hazard ratios (HRs and aHRs) with their corresponding 95% confidence intervals (95% CIs) for the association of PID and ovarian cancer risk, with and without adjusting for potential confounders. All models were adjusted for age and comorbidities. All statistical analyses were performed using SAS software version 9.4 (SAS Institute, Cary, NC, USA). A *P*-value of < 0.05 was considered to denote a statistically significant relationship.

## Results

Table 1 presents comparisons of demographic and clinical variables between the PID cohort and controls. The peak age of PID development was between 20 and 30 years (31.1%). Significantly more diagnoses of uterine cancer, endometriosis, and infertility were identified among the PID cases than among controls (all *P* < 0.001). Mean follow-up times were 9.44 ± 3.62 years and 9.24 ± 3.71 years for the PID cohort and controls, respectively.

**Table 1:** Baseline variables for patients with PID and women without PID (controls)

	Patients with PID		Controls		*P* value*
	(*n* = 64,925)		(*n* = 129,850)		
	*n*	%	*n*	%	
Age, years					> 0.99
< 20	4529	6.98	9058	6.98	
20–30	20,215	31.1	40,430	31.1	
30–40	18,739	28.8	37,478	28.8	
40–50	14,073	21.6	28,146	21.6	
50–60	4866	7.49	9732	7.49	
≥ 60	2503	3.86	5006	3.86	
Mean (SD)^†^	35.43 (12.1)		35.38 (12.3)		0.37
Comorbidity					
Lynch syndrome and colon cancer	69	0.11	108	0.08	0.11
Breast cancer	257	0.40	538	0.41	0.54
Uterine cancer	66	0.10	67	0.05	< 0.0001
Rectum cancer	52	0.08	80	0.06	0.13
Endometriosis	4,241	6.53	2,552	1.97	< 0.0001
Infertility	3,455	5.32	3,263	2.51	< 0.0001
Obesity	524	0.81	860	0.66	0.0003
Monthly income (NTD)					< 0.0001
< 15,000	31,211	48.0	64,575	49.7	
15,000–29,999	27,453	42.3	50,240	38.7	
≥ 30,000	6,261	9.64	15,035	11.6	

*PID* pelvic inflammatory disease, *SD* standard deviation, *NTD* New Taiwan dollar

^*^P values were determined using the Chi-square test for comparisons between women with PID and controls

^†^Mean follow-up times were 9.44 years (SD, 3.62) for the PID cohort and 9.24 years (SD, 3.71) for controls

**Fig. 2:**
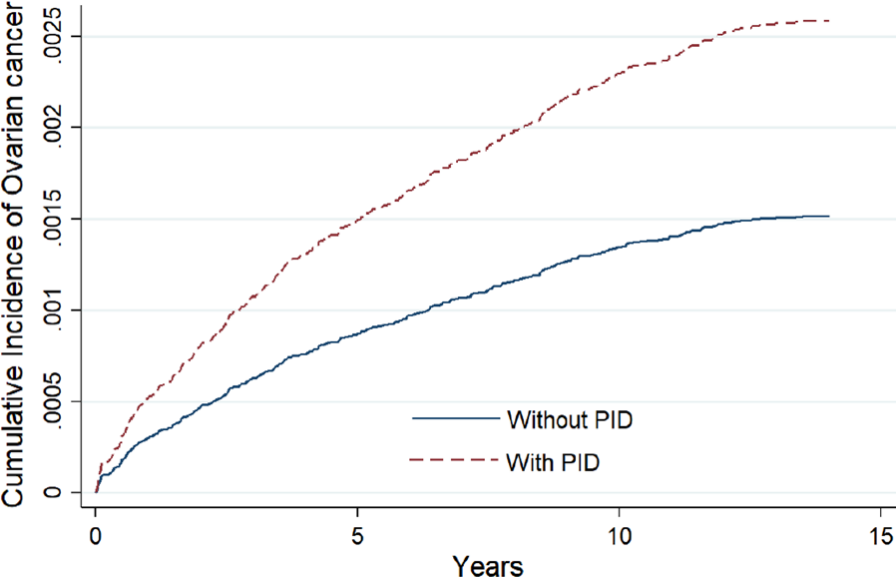
Using Aalen-Johansen survival statistics, it showed crude overall survival curves by with and without pelvic inflammatory disease. Over follow-up, the cumulative incidence of ovarian cancer was significantly higher in the PID cohort than among controls. (log-rank P < 0.0001)

Incidence rates of ovarian cancer per 1,000 person-years were 0.27 in the PID cohort and 0.16 in the control cohort (Table 2). The risk of ovarian cancer was significantly higher among women with PID than among those without in both unadjusted (HR, 1.67; 95% CI, 1.36–2.05; *P* < 0.001) and adjusted models (aHR, 1.49; 95% CI, 1.21–1.84; *P* < 0.001). Several comorbidities were significantly more common among women with PID than among those without, including Lynch syndrome and colon cancer (aHR, 5.47; 95% CI, 1.48–20.1; *P* < 0.05), breast cancer (aHR, 1.93; 95% CI, 1.03–5.23; *P* < 0.05), uterine cancer (aHR, 13.9; 95% CI, 5.70–34.2; *P* < 0.001), endometriosis (aHR, 2.93; 95% CI, 2.09–4.12; *P* < 0.001), and infertility (aHR, 1.84; 95% CI, 1.21–2.80; *P* < 0.01). Over follow-up, the cumulative incidence of ovarian cancer was significantly higher in the PID cohort than among controls (Log-rank test, *P* < 0.0001) (Fig. 2).

**Table 2:** Incidence and crude and adjusted risks of ovarian cancer according to age, comorbidity and monthly income for women with PID and those without (controls)

	Ovarian cancer			Crude HR (95% CI)	Adjusted HR^†^ (95% CI)
	Event	PY	IR		
Pelvic inflammatory disease	168	613,316	0.27	1.67 (1.36–2.05)***	1.49 (1.21–1.84)***
Age, years					
< 20	11	123,291	0.08	1 (reference)	1 (reference)
20–30	62	559,564	0.11	1.24 (0.65–2.36)	1.16 (0.61–2.22)
30–40	109	545,092	0.19	2.26 (1.21–4.20)**	1.98 (1.04–3.78)*
40–50	121	403,108	0.30	3.38 (1.82–6.27)***	3.01 (1.57–5.75)***
50–60	42	125,894	0.33	3.71 (1.91–7.21)***	3.32 (1.66–6.64)***
≥ 60	20	57,330	0.34	3.82 (1.83–7.97)***	3.31 (1.54–7.10)**
Comorbidity					
Lynch syndrome and colon cancer	4	1200	3.33	16.1 (6.01–43.1)***	5.47 (1.48–20.1)*
Breast cancer	4	6104	0.65	3.20 (1.19–8.57)*	1.93 (1.03–5.23)*
Uterus cancer	5	832	6.00	25.5 (11.8–69.0)***	13.9 (5.70–34.2)***
Rectum cancer	3	864	3.47	16.6 (5.33–51.7)***	3.25 (0.72–14.6)
Endometriosis	41	54,969	0.74	3.97 (2.87–5.49)***	2.93 (2.09–4.12)***
Infertility	25	59,497	0.42	2.15 (1.43–3.23)***	1.84 (1.21–2.80)**
Obesity	3	10,516	0.28	1.38 (0.44–4.30)	1.09 (0.35–3.40)
Monthly income (NTD)					
< 15,000	130	836,290	0.15	1 (reference)	1 (reference)
15,000–29,999	189	763,730	0.24	1.62 (1.29–2.02)***	1.13 (0.89–1.43)
≥ 30,000	46	214,260	0.21	1.40 (1.00–1.97)*	0.98 (0.69–1.39)

*PID* pelvic inflammatory disease, *HR* hazard ratio, *PY* person-years, *IR* incidence rate per 1000 person-years, *CI* confidence interval, *NTD* New Taiwan dollar

^†^HR adjusted for age, Lynch syndrome and colon cancer, breast cancer, uterus cancer, rectum cancer, endometriosis, infertility, obesity, and monthly income

^*^P < 0.05, **P < 0.01, ***P < 0.001, versus the reference group

The incidence and HR of of ovarian cancer in populations with or without PID relative to those of the controls are listed in Additional file 1: Table S. Despite the other factors, patients with a history of PID had HRs for ovarian cancer ranging from 1.37 (P < 0.05) to 5.06 (P < 0.001), which were significantly different compared with the values of those without a history of PID.

## Discussion

International investigations, including cohort studies, have implied some association between PID and ovarian cancer [6, 7, 24, 25]. This large-scale nationwide population-based study shows that compared with women without PID, women with PID are at higher risk of developing ovarian cancer and that this risk increases over time. Another large nationwide population-based cohort study suggests that there is no increased risk for ovarian, breast or uterine cancer among women with PID [8], in contrast to other researchers, who have described an increased risk of serous borderline ovarian tumors, but not of the mucinous type, in women with a history of PID[26]. Likewise, Rasmussen and colleagues have reported finding a positive association between low-grade, borderline ovarian tumors and PID in a pooled analysis of evidence from 13 case–control studies conducted between 1989 and 2009 [7], and a large Italian case–control study performed between 1983 and 1991 has shown an increased risk of ovarian cancer in women with a history of PID [12]. Interestingly, a meta-analysis of studies stratified by race has found a significant positive association between PID and ovarian cancer in studies conducted among Asian women, but only a slightly significant association amongst Caucasians [27]. One population-based cohort study that included 34 years of follow-up data from all women in Denmark (*n* = 1,318,929) showed a statistically significantly increased risk of serous ovarian cancer in women with a PID history, although there was no statistically significant relationship between PID and overall ovarian cancer [6].

Evidence from two independent case–control studies supports the association between PID and ovarian cancer, showing that antibodies against *Chlamydia trachomatis* infection may double the risk of ovarian cancer [28]. One research group has suggested that not only does PID increase the risk for developing ovarian cancer, but also that the risk increases with the number of PID episodes [29]. A more recent study has reported that the risk of high-grade serous ovarian cancer was increased in women with a diagnosis of PID and also in those with a personal history of breast cancer [30]. Interestingly, that study found that the risk for developing high-grade serous ovarian cancer reduced with increased parity, but not in women with a personal history of breast cancer[30]. There are no large-scale, long-term follow-up studies that have examined the relationship between previous PID and ovarian cancer in Taiwan or other Asian countries. Although one research group from Taiwan has reported finding a significant association between PID and ovarian cancer, the 3-year follow-up is probably insufficient time to accurately determine cancer formation [25].

The evidence from our large nationwide study with approximately 10 years of follow-up reveals that PID is a significant risk factor for ovarian cancer and that women aged 40 years and older with PID are at greater risk of developing ovarian cancer than younger-aged women. An increased risk of ovarian cancer associated with PID supports the hypothesis that inflammatory processes may contribute to the etiology of ovarian cancer [30, 31]. From our result, there is a big jump up in the cumulative incidence in the PID group immediately at the start of follow up. This indicates that at least in some cases, PID may appear to be a symptom of ovarian cancer or that a previously undiagnosed cancer is found upon examination for PID. Since women with a history of PID appear to be at high risk for ovarian cancer, screening these women for ovarian cancer may be an effective way to detect and treat the disease early. Prophylactic salpingo-oophorectomy is recommended for the prevention of ovarian cancer in high-risk women; there is no proven overall survival benefit in women with average risk for ovarian cancer, in whom the procedure has been associated with a significant increase in mortality from cardiovascular disease [32, 33]. Prophylactic hysterectomy with bilateral salpingo-oophorectomy has proven to be an effective way of preventing ovarian cancer in women with the Lynch syndrome [34].

Our study also reveals an increased risk of ovarian cancer in women with histories of endometriosis, infertility, Lynch syndrome, or breast cancer, in line with evidence from large cohort studies. For instance, the risk of epithelial ovarian cancer in women with a history of endometriosis is around 1.3–1.9 times higher than the risk among women without any such history [35, 36] The lifetime risk of ovarian cancer is 10–12% in women with the Lynch syndrome [34]. A multinational pooled analysis of infertility and fertility drug use in eight ovarian cancer case–control investigations reported that infertility increases the overall risk of ovarian cancer, whereas no association was observed between use of ovulation-inducing medications and ovarian cancer [37]. Other researchers have also reported that infertility alone is an independent risk factor for the development of ovarian cancer [38]. Finally, a personal history of breast cancer has been associated with a 3-fold increase in the rate of high-grade serous ovarian cancer [30].

Prevention of ovarian cancer is an important issue. Our study data are useful for clinicians wishing to counsel patients about the prevention and treatment of PID. The prevention of PID, including sex education and information, and the promotion of condom use, is an important issue for infectious diseases as well as future cancer risk [1]. Our study findings show that among important risk factors for ovarian cancer, the most important was PID.

The strengths of our study include its population-based design, the use of well-established cohort data, a large nationwide sample and an approximate 10-year follow-up period that identifies PID as a crucial risk factor in the development of ovarian cancer. Some limitations must be noted with this study. First, the NHIRD database does not code for the severity of PID, nor does it provide details of the pathogenesis and microbiology of PID, what types of infertility, or details about the cell types of ovarian cancers. Thus, we were unable to analyze any details concerning the pathophysiological relationship between ovarian cancer and PID. Second, the NHIRD registry lacks detailed records about laboratory results, parity, health-related habits or personal histories of patients, such as oral contraception use, family history and gynecological history, some of which are recognized risk factors for ovarian cancer. Third, the general incidence of PID may be underestimated if calculations fail to account for women with subclinical PID, or if women with PID fail to make a hospital visit due to personal or cultural reasons [39]. Fourth, this study was based on inpatient and outpatient data on the NHIRD database. Although the annual coverage rate of the NHI program has been estimated at 99.6%, under lack of 100% coverage, some bias may still exist of the general population. Fifth, comorbidities that happen prior to diagnosis of PID but after entry to the cohort study was not able to involved in this study. Sixth, the mean follow-up time is about 10 years, some of the ovarian cancer happened at advanced age might be miss.

## Conclusions

This study revealed that PID is an independent and significant risk factor for ovarian cancer. Compared with women without PID disease, those with a history of PID had a 1.49-fold higher risk of developing ovarian cancer (*P* < 0.001). More international study data are needed to verify this finding in our Taiwanese population. Our findings suggest that gynecologists should undertake careful assessments and closely follow patients with PID, who are at long-term risk of developing ovarian cancer.

## Supplementary Information

**Additional file 1: Table S.** Incidence rates and hazard ratios of ovarian cancer between the PID cohort and controls, stratified by age, comorbidities and monthly income.

## Data Availability

The data that support the findings of this study are available from the National Health Insurance Research Database (NHIRD) in Taiwan, but restrictions apply regarding the availability of these data, which were used under license for the current study and thus are not publicly available. The data are, however, available from the authors upon reasonable request and with permission of the National Health Insurance Research Database (NHIRD) in Taiwan.

## Abbreviations

PID: Pelvic inflammatory disease
NHIRD: National Health Insurance Research Database
LHID: Longitudinal Health Insurance Database
HRs: Hazard ratios

## References

[1] Workowski KA, Bolan GA (2015). Sexually transmitted diseases treatment guidelines (2015). Reprod Endocrinol.

[2] Toth M (1993). Pregnancy outcome following pelvic infection. Infect Dis Obstet Gynecol.

[3] Westrom L (1995). Effect of pelvic inflammatory disease on fertility. Venereology.

[4] Bender N (2011). Chlamydia infection, pelvic inflammatory disease, ectopic pregnancy and infertility: cross-national study. Sex Transm Infect.

[5] Bychkov V (1990). Ovarian pathology in chronic pelvic inflammatory disease. Gynecol Obstet Investig.

[6] Rasmussen CB (2017). Is pelvic inflammatory disease a risk factor for ovarian cancer?. Cancer Epidemiol Biomarkers Prev.

[7] Rasmussen CB (2017). Pelvic inflammatory disease and the risk of ovarian cancer and borderline ovarian tumors: a pooled analysis of 13 case–control studies. Am J Epidemiol.

[8] Shen CC (2016). Risk of uterine, ovarian and breast cancer following pelvic inflammatory disease: a nationwide population-based retrospective cohort study. BMC Cancer.

[9] Goff BA (2004). Frequency of symptoms of ovarian cancer in women presenting to primary care clinics. JAMA.

[10] Rossing MA (2010). Predictive value of symptoms for early detection of ovarian cancer. J Natl Cancer Inst.

[11] Doubeni CA, Doubeni AR, Myers AE (2016). Diagnosis and management of ovarian cancer. Am Fam Phys.

[12] Parazzini F (1996). Pelvic inflammatory disease and risk of ovarian cancer. Cancer Epidemiol Biomarkers Prev.

[13] Risch HA, Marrett LD, Howe GR (1994). Parity, contraception, infertility, and the risk of epithelial ovarian cancer. Am J Epidemiol.

[14] Hartge P (1989). A case-control study of epithelial ovarian cancer. Am J Obstet Gynecol.

[15] Franceschi S (1982). Risk factors for epithelial ovarian cancer in Italy. Am J Epidemiol.

[16] Charbonneau B (2013). The immune system in the pathogenesis of ovarian cancer. Crit RevTM Immunol.

[17] Tsai M-S (2017). Chang Gung Research Database: a multi-institutional database consisting of original medical records. Biomed J.

[18] Burkman RT (2002). Reproductive hormones and cancer: ovarian and colon cancer. Obstet Gynecol Clin N Am.

[19] Abu-Rustum NR, Barakat RR, Curtin JP (1997). Ovarian and uterine disease in women with colorectal cancer. Obstet Gynecol.

[20] Mori M (1988). Reproductive, genetic, and dietary risk factors for ovarian cancer. Am J Epidemiol.

[21] Wooster R, Weber BL (2003). Breast and ovarian cancer. N Engl J Med.

[22] Melin A (2006). Endometriosis and the risk of cancer with special emphasis on ovarian cancer. Hum Reprod.

[23] Chu NF (2005). Prevalence of obesity in Taiwan. Obes Rev.

[24] Coussens LM, Werb Z (2002). Inflammation and cancer. Nature.

[25] Lin HW (2011). Risk of ovarian cancer in women with pelvic inflammatory disease: a population-based study. Lancet Oncol.

[26] Rasmussen CB (2016). Increased risk of borderline ovarian tumors in women with a history of pelvic inflammatory disease: A nationwide population-based cohort study. Gynecol Oncol.

[27] Zhou Z (2017). Pelvic inflammatory disease and the risk of ovarian cancer: a meta-analysis. Cancer Causes Control.

[28] Trabert B (2019). Antibodies against chlamydia trachomatis and ovarian cancer risk in two independent populations. J Natl Cancer Inst.

[29] Risch HA, Howe GR (1995). Pelvic inflammatory disease and the risk of epithelial ovarian cancer. Cancer Epidemiol Biomarkers Prev.

[30] Stewart LM (2018). Risk of high-grade serous ovarian cancer associated with pelvic inflammatory disease, parity and breast cancer. Cancer Epidemiol.

[31] Zardawi IM (2014). Primary fallopian tube carcinoma arising in the setting of chronic pelvic inflammatory disease. Case Rep Med.

[32] Lipschutz DI (2013). Long-term mortality associated with oophorectomy compared with ovarian conservation in the Nurses' Health Study. Obstet Gynecol.

[33] Evans EC (2016). Salpingo-oophorectomy at the time of benign hysterectomy: a systematic review. Obstet Gynecol.

[34] Schmeler KM (2006). Prophylactic surgery to reduce the risk of gynecologic cancers in the Lynch syndrome. N Engl J Med.

[35] Park HK (2018). Benign gynecologic conditions are associated with ovarian cancer risk in African-American women: a case-control study. Cancer Causes Control.

[36] Somigliana E (2006). Association between endometriosis and cancer: a comprehensive review and a critical analysis of clinical and epidemiological evidence. Gynecol Oncol.

[37] Ness RB (2002). Infertility, fertility drugs, and ovarian cancer: a pooled analysis of case-control studies. Am J Epidemiol.

[38] Wallach EE, Bristow RE, Karlan BY (1996). Ovulation induction, infertility, and ovarian cancer risk. Fertil Steril.

[39] Wiesenfeld HC (2012). Subclinical pelvic inflammatory disease and infertility. Obstet Gynecol.

