# Supplementary material for: Association of pelvic inflammatory disease (PID) with ovarian cancer: a nationwide population-based retrospective cohort study from Taiwan
Source: BMC Womens Health. 2021 Jul 28;21:274. doi: 10.1186/s12905-021-01413-2 (PMC8320223; doi:10.1186/s12905-021-01413-2)
Supplement: Supplementary file 1 — Additional file 1: Table S. Incidence rates and hazard ratios of ovarian cancer between the PID cohort and controls, stratified by age, comorbidities and monthly income. [file 12905_2021_1413_MOESM1_ESM.docx]

**Table S.** Incidence rates and hazard ratios of ovarian cancer between the PID cohort and controls, stratified by age, comorbidities and monthly income

|  | **Pelvic inflammatory disease** | | | | | | | | **Crude HR (95% CI)** | **Adjusted HR (95% CI)** |
| --- | --- | --- | --- | --- | --- | --- | --- | --- | --- | --- |
|  | **No** | | | | | **Yes** | | |  |  |
|  | **Event** | **PY** | | | **IR** | **Event** | **PY** | **IR** |  |  |
| **Age** |  |  | | |  |  |  |  |  |  |
| <20 | 5 | 82,052 | | | 0.06 | 6 | 41,239 | 0.14 | 2.38 (0.72–7.82) | 1.88 (0.54–6.50) |
| 20–30 | 33 | 369,042 | | | 0.08 | 29 | 190,522 | 0.15 | 1.70 (1.03–2.80)* | 1.44 (0.86–2.42) |
| 30–40 | 56 | 360,149 | | | 0.15 | 53 | 184,943 | 0.28 | 1.84 (1.26–2.68)** | 1.56 (1.06–2.30)* |
| 40–50 | 72 | 267,908 | | | 0.26 | 49 | 135,200 | 0.36 | 1.35 (0.93–1.94) | 1.22 (0.84–1.77) |
| 50–60 | 21 | 83,717 | | | 0.25 | 21 | 42,177 | 0.49 | 1.99 (1.08–3.64)* | 1.94 (1.05–3.58)* |
| ≥60 | 10 | 38,094 | | | 0.26 | 10 | 19,236 | 0.51 | 1.99 (0.83–4.80) | 2.11 (0.86–5.12) |
| **Comorbidity** |  |  | | |  |  |  |  |  |  |
| Lynch syndrome and colon cancer | | |  |  |  |  |  |  |  |  |
| No | 194 | 120,0238 | | | 0.16 | 167 | 612,841 | 0.27 | 1.69 (1.37–2.07)*** | 1.51 (1.22–1.86)*** |
| Yes | 3 | 724 | | | 4.14 | 1 | 476 | 2.10 | 0.54 (0.05–5.28) | 0.51 (0.05–5.23) |
| Breast cancer |  |  | | |  |  |  |  |  |  |
| No | 196 | 1,196,916 | | | 0.16 | 165 | 611,259 | 0.26 | 1.65 (1.34–2.03)*** | 1.47 (1.19–1.81)*** |
| Yes | 1 | 4,046 | | | 0.24 | 3 | 2,058 | 1.45 | 5.70 (0.59–54.8) | 6.43 (0.66–62.3) |
| Uterine cancer |  |  | | |  |  |  |  |  |  |
| No | 194 | 1,200,519 | | | 0.16 | 166 | 612,928 | 0.27 | 1.68 (1.36–2.06)*** | 1.50 (1.21–1.86)*** |
| Yes | 3 | 443 | | | 6.77 | 2 | 389 | 5.14 | 0.76 (0.12–4.57) | 1.30 (0.16–10.3) |
| Rectum cancer |  |  | | |  |  |  |  |  |  |
| No | 195 | 1,200,418 | | | 0.16 | 167 | 612,996 | 0.27 | 1.68 (1.36–2.06)*** | 1.50 (1.21–1.85)*** |
| Yes | 2 | 544 | | | 3.67 | 1 | 321 | 3.11 | 0.81 (0.07–8.95) | 4.86 (0.21–107.9) |
| Endometriosis |  |  | | |  |  |  |  |  |  |
| No | 186 | 1,180,823 | | | 0.15 | 138 | 578,487 | 0.23 | 1.51 (1.21–1.89)*** | 1.47 (1.17–1.83)*** |
| Yes | 11 | 20,139 | | | 0.54 | 30 | 34,830 | 0.86 | 1.58 (0.79–3.15) | 1.56 (0.78–3.15) |
| Infertility |  |  | | |  |  |  |  |  |  |
| No | 193 | 1,172,666 | | | 0.16 | 147 | 582,116 | 0.25 | 1.53 (1.24–1.90)*** | 1.40 (1.12–1.74)** |
| Yes | 4 | 28,296 | | | 0.14 | 21 | 31,201 | 0.67 | 4.73 (1.62–13.7)** | 5.06 (1.70–15.0)** |
| Obesity |  |  | | |  |  |  |  |  |  |
| No | 196 | 1,194,412 | | | 0.16 | 166 | 609,350 | 0.27 | 1.66 (1.35–2.04)*** | 1.49 (1.21–1.84)*** |
| Yes | 1 | 6,550 | | | 0.15 | 2 | 3,966 | 0.50 | 3.30 (0.30–36.4) | 1.77 (0.15–20.6) |
| **Monthly income (NTD)** |  |  | | |  |  |  |  |  |  |
| <15,000 | 68 | 563,461 | | | 0.12 | 62 | 272,828 | 0.22 | 1.88 (1.33–2.65)*** | 1.65 (1.16–2.36)** |
| 15,000–29,999 | 103 | 486,391 | | | 0.21 | 82 | 277,339 | 0.29 | 1.46 (1.10–1.95)** | 1.37 (1.02–1.83)* |
| ≥30,000 | 26 | 151,110 | | | 0.17 | 20 | 63,149 | 0.31 | 1.84 (1.02–3.30)* | 1.57 (0.86–2.85) |

Abbreviations: PID, pelvic inflammatory disease; HR, hazard ratio; PY, person-years; IR, incidence rate per 1,000 person-years; CI, confidence interval; NTD, New Taiwan dollar.

^†^HR adjusted for age, Lynch syndrome and colon cancer, breast cancer, uterus cancer, rectum cancer, endometriosis, infertility, obesity, and monthly income.

| *p<0.05, **p<0.01, ***p<0.001, vs the separate comparisons |
| --- |
